# Risk factors for infection in older adults who receive home healthcare and/or home help: A protocol for systematic review and meta-analysis

**DOI:** 10.1097/MD.0000000000031772

**Published:** 2022-11-11

**Authors:** Ann E.M. Liljas, Janne Agerholm, Pär Schön, Bo Burström

**Affiliations:** a Department of Global Public Health, Karolinska Institutet, Stockholm, Sweden; b Institution for Social Work, Stockholm University, Stockholm, Sweden.

**Keywords:** protocol, systematic review, risk factors, infection, older adults, home healthcare, home help

## Abstract

The shift towards home-based care has resulted in increased provision of home healthcare and home help to older adults. Infections acquired in older adults while receiving home care have increased too, resulting in unplanned yet avoidable hospitalizations. In recent years, several studies have reported an array of factors associated with risk of infection; however, no previous systematic review has compiled such evidence, which is important to better protect older adults. Therefore, we have outlined the work of a systematic review that aims to identify risk factors for infection in older adults receiving home healthcare and/or home help.

**Methods:**

Searches for relevant studies will be conducted in five databases [MEDLINE, EMBASE (Excerpta Medica Database), Web of Science Core Collection, Cinahl (Cumulative Index to Nursing & Allied Health Literature) and Sociological Abstracts]. All types of studies will be included. Exposures considered refer to medical, individual, social/behavioral and environmental risk factors for infection (outcome). Two researchers will independently go through the records generated. Eligible studies will be assessed for risk of biases using the Cochrane risk of bias assessment tool and an overall interpretation of the biases will be provided. If the data allow, a meta-analysis will be conducted. It is possible that both quantitative and qualitative studies will be identified and eligible. Therefore, for the analysis, the Joanna Briggs Institute Reviewers’ Manual for mixed methods systematic reviews will be used as it allows for two or more single method reviews (e.g., one quantitative and one qualitative) to be conducted separately and then combined in a joint overarching synthesis.

**Results:**

The findings of the planned systematic review are of interest to healthcare professionals, caregivers, older adults and their families, and policy- and decisions makers in the health and social care sectors as the review will provide evidence-based data on multiple factors that influence the risk of infection among older adults receiving care in their homes.

**Conclusion:**

The results could guide future policy on effective infection control in the home care sector.

## 1. Introduction

The combination of more people living longer with chronic conditions and a shift towards home-based care has resulted in increased provision of healthcare and social care to older adults in their homes in many developed countries.^[1,2]^ Concurrently, in some countries such as the United States, the number of reported rates of infections developed in older adults while receiving home care have increased too, and account for 17% of unplanned hospitalizations.^[3]^ The percentage of unplanned hospitalizations is lower in for example Sweden (9%), yet many of these hospitalizations could have been avoided.^[4]^

Earlier studies have shown an array of factors that influence the risk of infection among older adults receiving care in their homes including individual factors such as older age, previous hospitalization, and poor health,^[5]^ a history of prior infections.^[6]^ and poor cognition.^[7]^ Further, in a recent qualitative study with home healthcare nurses, the nurses reported that their patients’ knowledge of and attitudes towards infection prevention and engagement in hygiene practices influenced the patients’ behaviors.^[8]^ Additionally, behaviors, beliefs and attitudes have been reported to affect the risks of infection among older adults with home healthcare. This includes lack of knowledge and understanding of their illness, hygiene practices and behaviors that prevent infections.^[9]^ The behaviors of those providing home care, often nurse assistants and informal caregivers, including good hand hygiene and use of sterile equipment have also been shown to influence the risk and spread of infection.^[10]^ Home healthcare staff have furthermore reported on environmental factors such as clutter, poor lighting, uncleanliness, and pets.^[8]^ Such barriers can negatively affect patient’s infection risk through increased stress levels in nurses and caregivers which may inhibit their adherence to properly follow infection prevention practices.^[11,12]^

In 2014, a systematic review on prevalence of infections and risk factors in home health care was published,^[13]^ showing that intravenous line-associated (catheter-related) infections (n = 19 out of 25 studies; 76%) were the most common types of infection. In the review, the most common infection risk factor was patient’s underlying medical conditions, reported in 8 out of 14 studies reporting on risk factors. Only one risk factor identified was non-medical (lower socio-economic status). Since 2014 when Shang and colleagues published their systematic review on risk for infection, a substantial amount of research on infection risk factors has been conducted, of which some studies are referred to in the previous paragraph above. Many of these recent studies have examined risks for infection in the home setting beyond medical factors e.g. behavioral or environmental factors, which are important aspects for infection risks that need to be taken into consideration. Additionally, contrary to the plans for the proposed systematic review, Shang and colleagues have neither reported on qualitative studies nor conducted a meta-analysis.

Several research studies published in the last few years have reported on multiple factors for the risk of infection in older adults with home care, however, evidence from these studies has not recently been compiled and systematically reviewed. This is needed to provide a better understanding of factors that are particularly important for the risk of infection in the home care setting. The importance of better protecting older adults with home care from infections and the potential consequences including hospitalization and death, has become apparent during the COVID-19 pandemic. The pandemic has affected especially older adults and presented severe challenges in preventing spread of infection including protecting older adults who are dependent on care providers. This emphasizes the need of compiled evidence of what factors particularly influence the risk of infections such as COVID-19 to older adults with home care. Novelty of the study includes the broad range of factors considering not only medical but also individual, social/behavioral and environmental factors. Novelty also involves the inclusion of home help. The systematic review on infection risk from 2014 showed that previous studies on home care have mainly focused on home healthcare,^[13]^ however infection risk is also highly relevant to home help as such care involves close contact too.^[8]^ Thus, when investigating infection risk in older adults who live at home it is essential to include home help. For the planned systematic review, home help refers to help received by professional or informal caregivers to manage everyday life.

## 2. Methods

### 2.1. Study aim

The aim is to identify risk factors for infection in older adults receiving home healthcare and/or home help.

### 2.2. Design and study registration

This study is a mixed-method systematic review. Additionally, a meta-analysis will be undertaken if the data reported in the eligible studies allow for it.

The development of the study included online searches for existing systematic reviews on the same topic including PubMed, PROSPERO, ResearchGate and Google to ensure that the planned study has not already been undertaken or is currently conducted. Then the study was outlined and registered with PROSPERO (CRD42021261159). The standard protocol checklist for systematic reviews, the Preferred Reporting Items for Systematic Reviews and Meta-Analyses Protocol, PRISMA-P, has been used for the planning of the study including the outline of this protocol (Appendix 1).

### 2.3. PICO (population, intervention/exposure, comparison, outcome)

To specify what data to be considered for the systematic review, a PICO process for the project has been developed and is presented below.

#### 2.3.1. *Population*.

Older adults (aged ≥ 65 years) who live in their own homes and receive healthcare by nurses and/or home help by formal/informal caregivers. Studies with populations of older adults who live in long-term institutions will be excluded.

#### 2.3.2. *Intervention/exposure*.

Measures of medical, individual, social/behavioral and environmental factors for risk of infection. Several of the exposures considered have been reported and identified in previous literature (see background). Medical factors considered include advanced age, poor health (e.g., frailty and ambulation), and previous hospitalizations. Individual factors considered refer to the older adult’s knowledge and understanding of their existing health problems/diseases, their hygiene practices and whether they have received influenza vaccination. Social/behavioral factors considered refer to those providing home care including whether the care is provided by professional or informal career, use of sterile equipment, tasks undertaken, and time (duration) spent with the older adult. For professional careers outcomes such as number of patients looked after per employee and geographical boundaries of the clients will be considered. Environmental factors considered are issues in the older adult’s home previously reported to directly or indirectly affect the risk of infection such as uncleanliness, clutter, poor lighting, and pets.

#### 2.3.3. *Comparison*.

No restriction.

#### 2.3.4. *Outcomes*.

Infection, infectious disease, communicable disease.

### 2.4. Search methods

**Figure 1.**
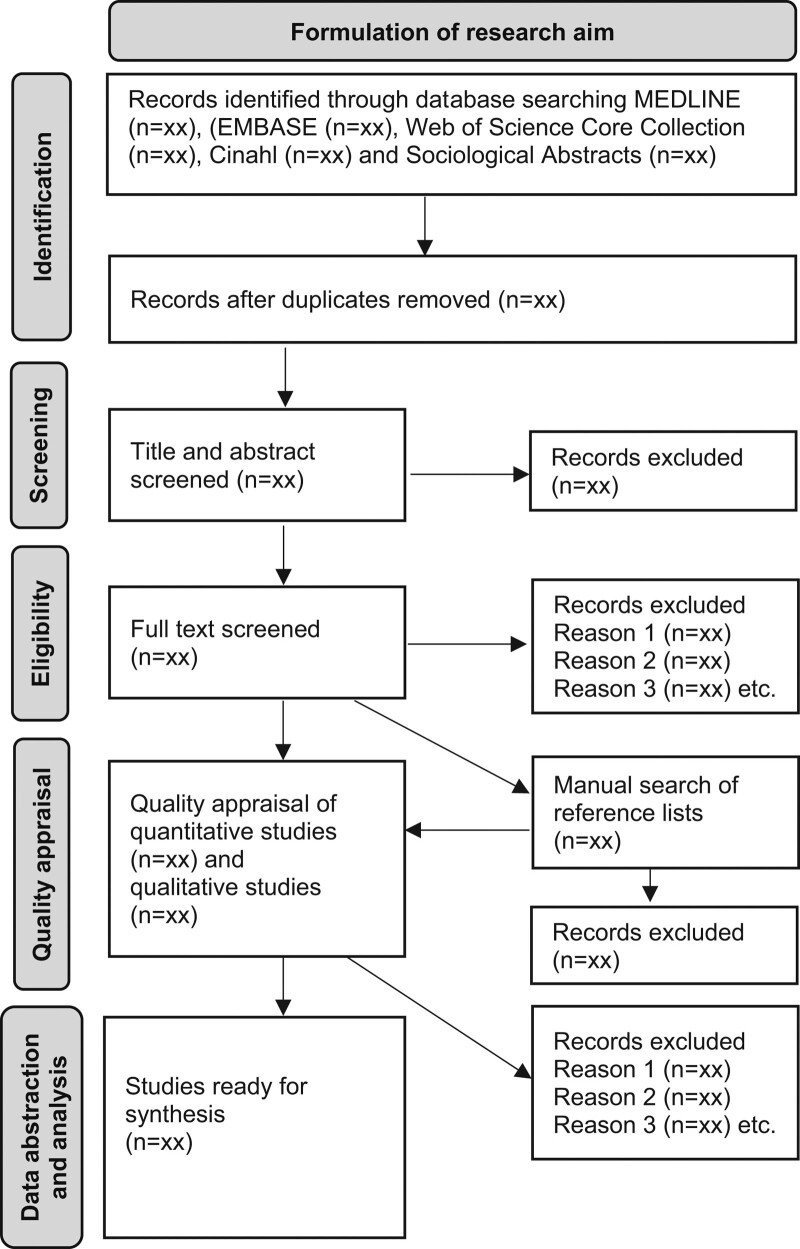
PRISMA flowchart of planned review. PRISMA = Preferred Reporting Items for Systematic Rev.

The search strategy has been outlined in a flow chart in Figure 1. Searches will be conducted in the databases MEDLINE, EMBASE (Excerpta Medica Database), Web of Science Core Collection, Cinahl (Cumulative Index to Nursing & Allied Health Literature) and Sociological Abstracts. Keywords considered for the searches include home care, home health care and home help in various combinations with infection, infectious diseases and communicable diseases. The search terms will be specified in collaboration with the university librarians who will conduct the database searches. Identified records from the database searches will be downloaded (or entered manually) to EndNote. Manual searches of the reference list of studies to be assessed for quality will also be undertaken.

Two researchers will go through all titles independently and, when uncertain whether to consider a record to be included, also read the abstract. Selected studies will then be read in full by the two researchers to identify eligible studies. The results of this process will be reported using the PRISMA flowchart.

### 2.5. Inclusion and exclusion criteria

Type of study designs to be included are quantitative, qualitative and mixed-method studies. Studies with no original data, for example, reviews and studies not exploring the risk of infection in older adults with home care will be excluded.

There will be no restriction in terms of years or language as publications in foreign languages with no title or abstract in English or Swedish yet potentially eligible, will be translated.

### 2.6. Quality appraisal

Studies meeting the eligibility criteria will be assessed for risk of biases using the guidelines of Cochrane risk of bias targeting seven items which refer to selection, performance, detection, attrition and reporting, and rated as high, low or unclear risk.^[14]^ The rating for each bias assessed in each study will be reported in a table. The two researchers who assess risk of bias will provide an overall interpretation of the biases, summarized by exposure. Any disagreements between the researchers over eligibility and bias rating will be resolved through discussions with a third researcher. Additionally, to strengthening the validity of the systematic review, publication bias across studies and selective reporting bias within studies will be assessed. Such assessment will be undertaken using the framework for assessing the risk of bias due to missing results in a synthesis as outlined in the Cochrane Handbook for Systematic Reviews (Chapter 13).^[14]^ Also, the assessment of the certainty of evidence for each exposure will be undertaken in accordance with GRADE (Grading of Recommendations Assessment, Development and Evaluation) and presented in table format. The bias rating and decisions on inclusion/exclusion based on the bias assessment, and the consequences of such decisions, will be explicitly reported.

### 2.7. Data extraction

Descriptive data will be extracted from each study included in the systematic review and entered into a table using MS Excel. This will include author, title, year of publication, country, study population including age, study design, study period, sample size, type of infection, type of home care (healthcare/home help), and results relevant for this systematic review. For examples of such results, see PICO above. If necessary, the data extraction table will be refined to include possible additional variables after having extracted data from the first few eligible studies.

### 2.8. Synthesis

The plan for the data synthesis includes mixed methods systematic review as both quantitative and qualitative studies might be eligible for the systematic review. For this, the Joanna Briggs Institute (JBI) Reviewers’ Manual for mixed methods systematic reviews^[15]^ is a suitable guideline as it allows for two or more individual, single method reviews to be conducted separately and then combined in a joint overarching synthesis. A statistician at the department where the main author works will provide input to the statistical process. The statistical software programme Stata will be used for the meta-analysis. If there are sufficient data to examine home healthcare and home help separately, subset analysis of the two will be carried out. If a meta-analysis cannot be carried out, the effect estimates will be summarized followed by a synthesis. For qualitative studies, a thematic approach will be used that involves identifying categories in the data, followed by comparisons between the studies. Once the single syntheses are completed, they will be combined in an overarching synthesis.

### 2.9. Ethics and dissemination

There are no requirements of ethical approval for this systematic review. The results will be disseminated in a peer-reviewed journal and presented at conferences.

## 3. Discussion

This systematic review will extract relevant articles from five well-known databases and manual searches to provide evidence-based data on various factors that influence the risk of infection among older adults receiving care in their homes. Identified studies will be presented according to quantitative and qualitative methods including possible meta-analysis of the quantitative findings.

This review has potential limitations. It is uncertain whether the studies report the prerequisite statistics needed for a meta-analysis, a problem reported in a previous systematic review on infection risks in older adults.^[13]^ It is further possible that there is large variety in what various studies consider risk factors for infection and infection prevention control. By extracting the data precisely, the readers will be able to compare the data in their context.

The findings of the planned systematic review are of interest to healthcare professionals and caregivers, older adults and their close relatives, as well as the health and social care sectors. Older adults in need of care form a particularly vulnerable segment of the population that is rapidly growing with increasing demands of healthcare and social care.^[16]^ Knowledge on how to better protect independently living older adults from infection is important not only for them but also to strengthening the care systems. Further, this systematic review focuses on both healthcare needs and social care needs, and the findings will add to existing knowledge of the close links between healthcare and social care. Such knowledge is important as combined health and social care needs are likely to increase as more people live longer. It is also of importance in the development of future policy on effective infection control in the home care sector.

## Author contributions

AL planned the systematic review and prepared the protocol manuscript. All authors have provided feedback on the manuscript.

**Conceptualization:** Ann EM Liljas.

**Funding acquisition:** Ann EM Liljas.

**Writing – original draft:** Ann EM Liljas.

**Writing – review & editing:** Bo Burström, Janne Agerholm, Pär Schön.

## References

[1] Landers S Madigan E Leff B. The future of home health care: a strategic framework for optimizing value. Home Health Care Manag Pract. 2016;28:262–78. 27746670 10.1177/1084822316666368 PMC5052697

[2] Mestheneos E. Ageing in place in the European Union. 2011. Available at: https://www.oldertenants.org.au/content/ageing-place-the-european-union [access date September 22, 2022].

[3] Shang J Larson E Liu J. Infection in home health care: results from national outcome and assessment information set data. Am J Infect Control. 2015;43:454–9. 25681302 10.1016/j.ajic.2014.12.017 PMC4437508

[4] Hallgren J Fransson EI Kåreholt I. Factors associated with hospitalization risk among community living middle aged and older persons: results from the Swedish Adoption/Twin Study of Aging (SATSA). Arch Gerontol Geriatr. 2016;66:102–8. 27281475 10.1016/j.archger.2016.05.005

[5] Shang J Russell D Dowding D. A predictive risk model for infection-related hospitalization among home healthcare patients. J Healthc Qual. 2020;42:136–47. 32371832 10.1097/JHQ.0000000000000214 PMC7477895

[6] Thomas S Karas JA Emery M. Meticillin-resistant *Staphylococcus aureus* carriage among district nurse patients and medical admissions in a UK district. J Hosp Infect. 2007;66:369–73. 17673333 10.1016/j.jhin.2007.05.004

[7] Shang J Wang J Adams V. Risk factors for infection in home health care: analysis of national outcome and assessment information set data. Res Nurs Health. 2020;43:373–86. 32652615 10.1002/nur.22053 PMC7418221

[8] Russell D Dowding D Trifilio M. Individual, social, and environmental factors for infection risk among home healthcare patients: a multi-method study. Health Soc Care Community. 2021;29:780–8. 33606903 10.1111/hsc.13321 PMC8084932

[9] Dowding D Russell D Trifilio M. Home care nurses’ identification of patients at risk of infection and their risk mitigation strategies: a qualitative interview study. Int J Nurs Stud. 2020;107:103617. 32446014 10.1016/j.ijnurstu.2020.103617 PMC7418527

[10] Higginson R. Infection control when delivering intravenous therapy in the community setting. Br J Community Nurs. 2017;22:426–31. 28862904 10.12968/bjcn.2017.22.9.426

[11] Adams V Song J Shang J. Infection prevention and control practices in the home environment: Examining enablers and barriers to adherence among home health care nurses. Am J Infect Control. 2021;49:721–6. 33157183 10.1016/j.ajic.2020.10.021 PMC8093314

[12] Gershon RRM Pogorzelska M Qureshi KA. Home health care patients and safety hazards in the home: preliminary findings. Henriksen K Battles J Keyes M, eds. In: Advances in patient safety: New directions and alternative approaches. Vol. 1: Assessment. Rockville: Agency for Healthcare Research and Quality, 2008. 21249854

[13] Shang J Ma C Poghosyan L. The prevalence of infections and patient risk factors in home health care: a systematic review. Am J Infect Control. 2014;42:479–84. 24656786 10.1016/j.ajic.2013.12.018 PMC4438760

[14] Higgins J Thomas J. Cochrane Handbook for Systematic Reviews of Interventions Version 6.3. 2022. Available at: https://training.cochrane.org/handbook/current [access date September 22, 2022].

[15] Joanna Briggs Institute (JBI). Reviewers’ Manual for mixed methods systematic reviews. 2014. Available at: https://nursing.lsuhsc.edu/JBI/docs/ReviewersManuals/Mixed-Methods.pdf [access date 22 September 2022].

[16] Cristea M Noja GG Stefea P. The impact of population aging and public health support on EU labor markets. Int J Environ Res Public Health. 2020;17:1439. 10.3390/ijerph17041439 PMC7068414 32102277

